# Orthopaedic Telemedicine Clinics: A Patient Satisfaction Survey of 522 Patients

**DOI:** 10.7759/cureus.73392

**Published:** 2024-11-10

**Authors:** Tithi Jain, Hasan Rahij, Zaib Hilal, Rahij Anwar

**Affiliations:** 1 Trauma and Orthopaedics, Medway Maritime Hospital, Gillingham, GBR; 2 Orthopaedics, Medway Maritime Hospital, Gillingham, GBR; 3 Trauma and Orthopaedics, Imperial College Healthcare NHS Trust, London, GBR; 4 Trauma and Orthopaedics, Spire Alexandra Hospital, London, GBR

**Keywords:** covid-19, orthopaedics, patient satisfaction, remote consultation, telemedicine

## Abstract

Background

The COVID-19 pandemic necessitated the rapid adoption of telemedicine across medical specialties, including orthopedics. This study aimed to assess patient satisfaction with orthopedic telemedicine consultations and analyze the efficiency and limitations of this approach during the pandemic.

Methods

A cross-sectional survey was conducted involving 522 patients who underwent telephone consultations for orthopedic care between May 2020 and January 2021. Consultations were performed by a single surgeon using the electronic software 'Proxima' and self-designed Microsoft Word templates for documentation. Patient satisfaction was assessed at the end of each consultation. Data on consultation duration, documentation time, the need for face-to-face follow-up, and the impact on surgical decision-making were collected.

Results

The overall patient satisfaction rate with telemedicine consultations was 77.9%. However, 65.3% of patients required subsequent face-to-face assessment, primarily for physical examination (48.2%) or discussion of investigation results (13.4%). Among 88 patients already listed for surgery, 38.6% chose to delay their operations due to COVID-19 concerns. The average telephone consultation duration was 4.9 minutes (range: 2-16 minutes), with 86.7% of clinical documentation completed in less than 5 minutes. Patients who had surgery and did not experience any complications exhibited the highest level of satisfaction at 92.3%.

Conclusions

The research we conducted provides evidence that telemedicine can be useful in the management of orthopedic patients, especially those who have undergone surgery. Nevertheless, continued face-to-face evaluation remains vital in orthopedics due to its high rates of necessity. The use of electronic templates and software for documentation proved to be highly efficient, significantly reducing the time required for clinical documentation and potentially allowing for more patient interactions. As health systems progress with advances made possible by pandemics and other future challenges, it is likely that an ideal combination of remote delivery via telemedicine and traditional means will be a safer, more effective, and efficient way of providing orthopedic services.

## Introduction

The COVID-19 pandemic manifested a seismic shift in global healthcare delivery, leading to punctual modifications that aimed at maintaining continuity of care with minimal infection risks [1]. Most countries shifted from traditional approaches to telemedicine, which had long been considered a possible solution to healthcare access inadequacies, and found themselves relying entirely on it for physicians and their patients during quarantine and lockdown periods [2]. Orthopedic medicine, which has traditionally been based on human feedback, experienced a unique challenge while transitioning to remote consultations [3].

It is not a new idea to use telemedicine in orthopedics; this approach has existed for many years, even before COVID. It has been studied primarily for follow-up treatments in rural areas [4]. However, during the pandemic era, the practice of conducting telemedicine clinics was widely adopted in every area of orthopedic care, including initial patient consultations and post-operative follow-ups [5]. Questions on effectiveness, patient satisfaction, and limitations regarding telemedicine with orthopedic patients arose because of such a quick turnaround.

According to Kruse CS et al. [6], telemedicine presents certain difficulties despite its evident benefits such as reduced travel times, minimized exposure threat, and possibly shorter waiting times. Some of these challenges include technology-related barriers, fears for the quality of remote assessments, and lack of personal examination [7]. In orthopedics, where physical examination and direct patient contact play vital roles in diagnosis and treatment planning, the limitations of telemedicine can be particularly pronounced.

By the end of July 2020, it became evident that assessing patient satisfaction was crucial to truly measure the effectiveness of telemedicine in healthcare delivery [8]. Furthermore, this study provides insights into telemedicine protocols, highlighting the reasons for necessary changes in telemedicine practices and identifying areas where in-person visits remain essential [8]. The time-saving nature of telemedicine makes it particularly significant, as healthcare professionals increasingly adopt this new mode of care delivery [9].

The primary goal of this study is to evaluate patient satisfaction with teleconsultations in orthopedic medicine during the COVID-19 pandemic and to analyze how electronic software and templated documentation facilitated the process. To gain insights into patients’ perspectives on remote orthopedic care, we surveyed 522 individuals who attended telemedicine consultations from May 2020 through January 2021. Moreover, we also looked at how many patients needed a follow-up face-to-face assessment, how well clinical records were maintained using pre-prepared templates, and the total time taken for telemedicine consultations.

​The results from this research will help expand the existing knowledge base about how telemedicine works and its challenges in treating various orthopedic conditions. Since post-pandemic healthcare systems are still wrestling with the implications of COVID-19, it is pertinent to understand the role of telemedicine in ensuring care continuity. This study seeks to explore ideal standards for combined usage of virtual consultation and physical examination during the management of musculoskeletal illnesses, which are best diagnosed through manual inspections.

## Materials and methods

​An retrospective evaluation of patient satisfaction with the use of orthopedic telemedicine was conducted through a cross-sectional survey design. A total of 522 patients who attended telemedicine clinics were enrolled in the study from May 2020 to January 2021.

​The telemedicine clinics were conducted by a single surgeon, the senior author of the study, within the Practice Plus Group elective orthopedic units. These units were previously NHS treatment centers located in Ilford and Gillingham. Having one surgeon conduct all consultations was decided upon to ensure consistency in the approach and minimize variation in patients’ experiences that could arise from different practitioners' styles.

​Under the pandemic, there were telephone consultations between patients and their physicians as required by social distancing guidelines. For efficient clinical documentation, electronic software called ‘Proxima’ and self-designed Microsoft Word letter templates were the main tools used in this study. Without any secretarial or administrative support, these tools allowed us to assess how efficient direct clinician documentation is in telemedical contexts.

At the end of every telemedicine consultation, standardized questions were orally asked to patients, indicating their level of contentment with this novel form of patient engagement. This enabled instantaneous reactions from patients as they had just experienced it. Responses were saved and later examined for general patient satisfaction rates. ​We also collected additional data on how willing they were to proceed with planned surgery, for example, among those who had already had some surgery listed before COVID-19 struck (totaling 88). The aim was to find out how much influence COVID-19 had on people's choices towards elective procedures.

​The research also monitored the number of patients who underwent face-to-face assessments after their initial telephone consultations. These follow-up appointments were classified according to the reason for the in-person visit, such as a physical examination or discussion of results from various tests (for instance, MRI scans and nerve conduction studies). ​Two main parameters were tracked during each consultation to evaluate telemedicine’s effectiveness: the time taken to finish clinical documentation using pre-prepared letters and the duration of every telephone call made. A review of these parameters provided insight into the efficiency of telemedicine appointments in orthopedics. ​The study design allowed for the identification of specific groups of patients who might have been more responsive to tele-consultations. Patients without wound problems or joint pain after surgery were mainly focused on, as initial observations indicated they would be more likely to comply with remote follow-up.

​Through this, the percentage calculations of key outcomes such as patient satisfaction, the need for face-to-face follow-up, and efficiency metrics were aimed at simply describing data analysis. Conclusions about how effective or limited telemedicine is in orthopedic care were drawn from the results. ​Patient data privacy was ensured, while oral consent was sought for involvement in the satisfaction survey as ethical considerations. This study was conducted in accordance with the institution's ethical protocols and in compliance with applicable data protection laws.

## Results

There were 522 individuals involved in the project, which is a relatively large sample size. An age range from 18 to 85 shows that adult patients were included, but the average age of 57.3 years implies that this group is generally older than other groups found in orthopedic practice. In terms of gender representation, female patients made up 54.2% of the sample. Overall, the patients evaluated showed a high satisfaction rate of 77.9% (407 out of 522), indicating that most of them were pleased with the telemedicine consultations offered to them. This data points to an acceptance of telemedicine by the society tasked with providing these services during the COVID-19 pandemic period (Table 1).

**Table 1:** Patient demographics and overall satisfaction.

Characteristics	Value
Total patients	522
Age range (years)	18-85
Mean age (years)	57.3
Gender (% female)	54.2%
Overall satisfaction with telemedicine	77.9%

​Of these 88 pre-listed patients, 61.4% (54 individuals) went ahead with their planned operations, while 38.6% (34 patients) decided to postpone surgery because of fears arising from COVID-19 pandemic issues. This data suggests that the COVID-19 pandemic had a profound effect on the decision to proceed with surgery. For orthopedic patients, there are indications that COVID-19-related anxiety could have been responsible for more than one out of every three who opted to delay. Table 2 below shows this clearly.

**Table 2:** Surgical decision-making for pre-listed patients.

Decision	Number of Patients	Percentage
Proceeded with surgery	54	61.4%
Delayed surgery due to COVID-19 concerns	34	38.6%
Total pre-listed patients	88	100%

It was found that about 48 percent (252 patients) needed a physical examination, which highlights the main limitation of telemedicine in orthopedics. Moreover, 13% (70 patients) required direct consultations. Notably, only 35% (181 patients) escaped without an onsite consultation. Therefore, these findings suggest that although telemedicine has proved to be useful, a great number of patients still need an assessment face-to-face; thus, emphasizing much on the significance of physical examinations during orthopedic care work. The need and significance of a physical examination during orthopedic care cannot be underestimated (Table 3).

**Table 3:** Need for face-to-face assessment after initial telemedicine consultation.

Reason for Face-to-Face Assessment	Number of Patients	Percentage
Physical examination needed	252	48.2%
Discussion of investigation results	70	13.4%
Other reasons	19	3.7%
No face-to-face assessment needed	181	34.7%
Total	522	100%

Telephone consultation was useful for 77.9% (407 patients). However, 22.0% preferred a physical examination. Although most patients were satisfied with telephone contact, a vast majority still required face-to-face assessment, which raises doubts about its universal role in the delivery of orthopedic care (Table 4).

**Table 4:** Patient satisfaction with telemedicine consultations.

Satisfaction Level	Number of Patients	Percentage
Satisfied	407	77.9%
Preferred face-to-face examination	115	22%
No opinion	0	0.1%
Total	522	100%

Telephone conversations tested from a call length of an average period of 4.9 minutes, ranging from two to sixteen minutes, indicate that most consultations were of short duration. This suggests that 86.7% of them completed their clinical documentation in under five minutes, highlighting the potential efficiency of their record-keeping process. These figures suggest that telemedicine consultations may offer time-saving benefits, potentially allowing for a higher volume of patient interactions compared to traditional in-person hospital visits within the same timeframe. Documentation of clinical notes using pre-designed templates seemed to be significantly time-efficient (4.2 minutes) (Table 5).

**Table 5:** Efficiency metrics for telemedicine consultations.

Metric	Value
Average telephone call duration	4.9 minutes
Range of call durations	2-16 minutes
Clinical documentation completed in <5 minutes	86.7%
Average documentation time	4.2 minutes

In those with no issues post-operatively, satisfaction scores were highest at 92.3%, suggesting telemedicine might be more suitable for routine follow-ups after surgery than for pre-operative assessment of patients, where they recorded lower scores of 73.1%. Table 6 stratifies the patient groups based on satisfaction rates and requirements. The satisfaction rate was noted to be below 70% in patients who required consultations for anything other than post-operative follow-up (Table 6).

**Table 6:** Satisfaction by patient type.

Patient Type	Satisfaction Rate
Post-operative patients with no issues	92.3%
Pre-operative patients	73.1%
Patients requiring further investigations	68.5%
Overall	77.9%

## Discussion

Because of our research on orthopedic telemedicine clinics during COVID-19, we have learned a lot about patient satisfaction, the effectiveness of remote consultations, as well as the challenges encountered when implementing telemedicine in orthopedic care. The paper aims to focus on these findings in relation to other studies with an eye towards future practice.

​Our investigation discovered that 77.9% of patients were generally satisfied with telemedicine consultations, which corroborates similar findings from other studies carried out during the pandemic. For example, according to Abel K et al., 96% of orthopedic patients reported being completely satisfied with their video consultations [10]. Similarly, Abeler et al. indicated that 93% of patients were satisfied with orthopedic follow-up via telemedicine [11]. The slightly lower rate of satisfaction observed in our study might be explained by our exclusive use of telephone consultations as opposed to video calls, which allow for more visual interaction. ​Our study proves that face-to-face examination is still the preferred option, which we believe is a major challenge for orthopedic telemedicine: physical examination matters for diagnosis and treatment planning purposes. Tanaka MJ et al. addressed the limitations of the “virtual orthopedic examination” and suggested ways to enhance its effectiveness [5]. To enhance both patient satisfaction and diagnostic accuracy in future telemedicine protocols, orthopedic practices should consider incorporating these recommendations.

​The high rate of 65.3% of patients requiring face-to-face assessment after initial telemedicine consultations has significant adverse effects on health economics, including resource wastage, duplication of work, and increased healthcare costs. This double-appointment approach not only strains healthcare systems by potentially doubling the resources required for these patients but also burdens patients with additional time commitments and possible travel costs. Implementing more effective triage protocols, such as better pre-consultation screening tools and enhanced remote examination techniques, could help mitigate these issues by more accurately identifying patients who truly need in-person assessments, thereby optimizing resource allocation and improving the overall efficiency of telemedicine practice. This rate is higher than some reported figures in the literature. For example, Gilbert AW et al. found that only 3% of patients required in-person visits after virtual fracture clinic appointments [12]. However, their study focused on a specific subset of orthopedic patients, whereas our study encompassed a broader range of orthopedic conditions. ​The high rate of further face-to-face assessments in our study highlights the limitations of telemedicine in orthopedics, particularly for new patient evaluations or complex cases. Buvik A et al., in their randomized controlled trial, found that telemedicine was non-inferior to in-person consultations for selected orthopedic patients but excluded patients requiring complex physical examinations [4]. Our results suggest that careful patient selection and triage are crucial for the effective implementation of telemedicine in orthopedic practice.

​The efficiency metrics from our research suggest that telemedicine can be time-efficient, especially when implemented with adequate tools and templates, as the average duration of a call took 4.9 minutes, and 86.7% of clinical documentation was completed within 5 minutes. Similar efficiencies were reported by Holmgren AJ et al. during the COVID-19 pandemic in primary care telemedicine visits [13]. However, it is key to acknowledge that our research was based on an operation conducted by a single, highly experienced surgeon; hence, there may be differences depending on the health provider or locality. ​A total of 38.6% of pre-listed patients chose to delay having surgery due to COVID-19 concerns, aligning with broader overall patterns observed during the period. For example, Hanreich C et al. reported a 25% decline in elective orthopedic surgery volume when hospitals were shut down [14]. Patients' decision to postpone elective surgery addresses communication ambiguities regarding safety measures and risk factors related to untimely treatment. ​Our finding of high postoperative patient satisfaction, 92.3% in patients who had no complications as compared to pre-operative ones at 73.1%, is consistent with other studies. When it comes to postoperative visitations for orthopedic patients, specifically telemedicine had very positive outcomes: Rizzi AM et al. found out that telemedicine played a crucial role in ensuring successful recovery processes [15].

​Nevertheless, besides providing valuable insights, our study bears limitations. Our study's limitations include its reliance on a single surgeon's practice, exclusive use of telephone consultations, and timing during the height of the COVID-19 pandemic, which may have inflated satisfaction rates due to heightened anxiety about in-person visits. As the pandemic subsides, patient preferences may shift, potentially reducing satisfaction with telemedicine across all groups, while the use of video calls might have proven more effective than telephone consultations alone. Additionally, our study did not explicitly address critical issues of data protection and security in telemedicine, which are becoming increasingly important as healthcare moves into the digital realm.

​In future investigations, video technology integration should be addressed alongside using patient-reported outcomes in telemedicine and comparing the long-term outcomes of telemedicine versus regular hospital management. Additionally, advanced clinical software systems incorporating artificial intelligence and voice recognition could lead to more efficient and effective telemedicine consultations.

## Conclusions

Our study provides evidence that telemedicine can be a useful adjunct in the delivery of orthopedic care. Nevertheless, continued face-to-face evaluation is vital in orthopedics due to improved satisfaction rates. As health systems progress with advances made possible by pandemics and other future challenges, it is likely that an ideal combination of remote delivery via telemedicine and traditional means will be a safer, more effective, and efficient way of providing orthopedic services. Incorporation of technology, including electronic documentation combined with training, may lead to efficient resource utilization and could also have a positive effect on health economics.
